# Culturally Informed Communication of Neonatal Death in Chinese Neonatal Intensive Care Units

**DOI:** 10.1001/jamanetworkopen.2026.5919

**Published:** 2026-04-09

**Authors:** Hongyu Zhao, Chenglei Hu, Jinyang Geng, Jiaxin Wang, Yi Lu, Ericka Waidley, Huiwen Xu, Josephine Hegarty, Pingting Zhu

**Affiliations:** 1 School of Nursing, Yangzhou University, Yangzhou, China; 2 Linfield-Good Samaritan School of Nursing, Linfield College, Portland, Oregon; 3 School of Nursing and Midwifery, Brookfield Health Sciences Complex, University College Cork, Cork, Ireland

## Abstract

**Importance:**

Given the uniquely sensitive context of neonatal death in China, neonatal intensive care unit (NICU) clinicians face considerable pressure as they are required to communicate this profound loss to parents in a culturally appropriate manner.

**Objective:**

To examine how NICU physicians and nurses communicate with parents about neonatal death, navigate cultural expectations in the process, and manage communication decisions across varied clinical scenarios.

**Design, Setting, and Participants:**

This qualitative study used semistructured interviews to collect data and a descriptive phenomenological approach to analyze interview data. The study was conducted with clinicians at 2 NICUs in 2 tertiary care hospitals in Yangzhou City, Jiangsu Province, China. Participants were enrolled through purposeful sampling and included physicians and nurses with prior experience with caring for neonates. Data were collected from September 2024 to May 2025 and were analyzed from October 2024 to June 2025.

**Main Outcomes and Measures:**

Experiences of informing families of neonatal death. In-depth, semistructured interviews were conducted at a rate of 1 participant per day and continued until data saturation was achieved. Data were analyzed using the Colaizzi 7-step phenomenological method.

**Results:**

A total of 24 individuals (10 physicians and 14 nurses; mean [SD] age, 39.2 [7.5] years; 20 females [83%]) participated in the study. Among participants, 20 (83%) were married with at least 1 child, and the mean (SD) length of service was 16.3 (8.1) years. All participants held a bachelor’s degree, were of Han Chinese ethnicity, and had no religious affiliation. Three main themes and 9 subthemes emerged from the interviews. The first theme was that fathers were prioritized over mothers as to whom to make the death disclosure. The subthemes were perceiving the father as the primary family decision-maker, encountering barriers to direct death disclosure to the mother, and being instructed to prioritize death disclosure to the father. The second theme was that death disclosure was handled sensitively, with the explicit term *death* avoided. Its subthemes were incrementally disclosing bad news, avoiding directly using death-related vocabulary, and using metaphors to explain medical terminology. The third theme was that postdisclosure procedures were handled exclusively in accordance with cultural norms. The subthemes were suppressing personal expressions of grief, being delegated full authority for handling the child’s remains, and adjusting disposition arrangements to safeguard familial reputation.

**Conclusions and Relevance:**

In this qualitative study of death disclosure, participating NICU medical staff disclosed death with sensitivity to traditional Chinese cultural norms. Insights from their experiences highlight key areas for improvement, including reducing stigma around neonatal death, developing culturally sensitive bereavement services, and exploring parental experiences and preferences.

## Key Points

**Question:**

What are the experiences of neonatal intensive care unit (NICU) medical staff regarding communicating about neonatal death with parents in China?

**Findings:**

In this qualitative study involving 24 NICU physicians and nurses, neonatal deaths were disclosed primarily to fathers, the term *death* was avoided and metaphors were used in communication with parents, and emotions were restrained. In most instances, the hospital and staff managed newborns’ remains to protect families’ reputations.

**Meaning:**

Findings of this study highlight key areas for improvement such as reducing stigma around neonatal loss, developing culturally sensitive bereavement services, and exploring parental experiences and preferences regarding neonatal death and postmortem care.

## Teaser

This qualitative study describes the experiences of physicians and nurses about communicating with parents about neonatal death at 2 neonatal intensive care units (NICUs) in 2 hospitals in Yangzhou City, Jiangsu Province, China.

## Introduction

According to the latest statistics from the National Bureau of Statistics of China, the national neonatal mortality rate was 2.8 per 1000 live births as of 2023.^1^ Research indicates that the death of a child is often more profoundly distressing than other forms of bereavement (such as the loss of a parent or spouse).^2^ Death of a child often leads to prolonged grief, anxiety, depression, and posttraumatic stress disorder in parents.^3,4,5^ Parents may even experience physical symptoms, such as weight loss and insomnia.^6^ Furthermore, families experiencing neonatal death typically exhibit reduced social engagement in the short term.^3,7^ Communicating a newborn’s death to families in acute distress is a major challenge for clinicians.

In the context of cancer care, the disclosure of death has evolved into a relatively standardized procedural framework, wherein physicians follow the SPIKES (Setting up, Perception, Invitation, Knowledge, Emotions with Empathy, and Strategy or Summary) protocol.^8^ The SPIKES protocol outlines the steps required in communicating death: setting up the conversation, assessing the family’s perception, clarifying how much detail the family wishes to receive, delivering the news of death using appropriate language, and addressing emotions with empathic responses.^8,9^

However, practices in China regarding the disclosure of death-related information differ from those in the West.^10,11,12^ Within the Chinese cultural context, discussions surrounding death have historically been regarded as taboo.^12,13^ Concurrently, collectivism is recognized as a core cultural belief among Chinese families.^14^ Consequently, the disclosure of medical information is viewed as a family-centered rather than an individual-centered process.^15,16^ In the unique context of neonatal death, fathers—as a senior family member—often assume the primary decision-making role.^17,18^ Meanwhile, mothers observe the traditional postpartum period known as *zuoyuezi*, which instructs them to adhere to certain taboos, manifested in restrictions on their living environment, behavior, and diet.^19,20,21,22,23^ These factors complicate effective communication between health care practitioners and family members.^24^

Current research primarily concentrates on enhancing clinical communication skills, such as disclosure protocols and empathy language training.^25^ However, it has been proposed that developing strategies for truth-telling must account for the critical roles of cultural sensitivity and family dynamics.^12,26^ Despite this recognition, there is a notable lack of comprehensive research exploring the lived communication experiences of clinicians in Chinese neonatal intensive care units (NICUs) as they engage with families during end-of-life care within the context of prevailing cultural norms.

This study adopted a descriptive phenomenological approach to capture health care professionals’ cognitive, emotional, and behavioral responses during the death disclosure process. Our objective was to examine how NICU physicians and nurses communicate with parents about neonatal death, navigate cultural expectations in the process, and manage communication decisions across varied clinical scenarios. The insights derived from their experiences could help inform future practice.

## Methods

### Study Design

We conducted semistructured interviews with clinicians working at 2 level-III NICUs in 2 tier-III, grade-A tertiary care hospitals in Yangzhou City, Jiangsu Province, China. The Yangzhou University Institutional Review Board approved this qualitative study. Written informed consent was provided by all participants. We followed the Consolidated Criteria for Reporting Qualitative Research (COREQ) reporting guideline.^27^

Participation was entirely voluntary. Participants had the right to withdraw from the study within 2 weeks of enrollment without any repercussions, and their data would be promptly destroyed. No participant withdrew consent. Audio files and transcription data were deidentified, encrypted, and securely stored, with access restricted to the research team. As an additional support measure, a check-in, follow-up telephone call assessing participants’ well-being was conducted within 1 month of the study start.^28^ A psychiatrist from our team was available to participants needing psychological support.

### Recruitment

Purposive sampling was used to ensure representation across varying levels of clinical experience. By incorporating differentiated criteria based on participants’ length of health care service, the study aimed to capture diverse professional perspectives. Physicians and nurses at the NICUs of 2 of China’s highest-grade tertiary care hospitals were recruited. An inclusion criterion was at least 1 prior experience of caring for neonates, including supporting families at the end of life.

Through the hospital information system, health care staff screened potential participants against the eligibility criteria and extracted the contact information of eligible clinicians. Potential participants were initially contacted via a text message detailing the study’s purpose and interview procedures. After a 1-week consideration period, we contacted potential participants by telephone to provide further study details and to ask if they would participate. After obtaining verbal consent from participants, we established communication via WeChat (Tencent Holdings Limited)—a common application that is mostly used as an alternative to telephone calls in China—to schedule the interview.

Interviews were conducted at a rate of 1 participant per day and continued until data saturation was achieved. Due to the sensitive nature of the topics, daily interviews were limited to safeguard interviewer well-being and ensure data integrity.

### Data Collection

The semistructured interview guide included open-ended questions aligned with the study objectives (Box). The guide was refined following expert consultation (P.Z. and J.H.) and 2 pilot interviews with 2 nurses from the NICU of a tertiary hospital. The initial interview questions were posed more broadly and then narrowed. Interview format (in person or by telephone) was determined by participant preference. Prior to each session, participants provided written informed consent, including permission for audio recording. In-person interviews were conducted in a quiet, private, and comfortable hospital setting. Interviews were conducted in Chinese by 2 researchers (H.Z. and J.G.) who have formal training in qualitative methodologies and interview techniques. Each session lasted 30 to 50 minutes, and field notes were taken during and immediately after the session. All interviews were audio recorded and transcribed on the same day they were conducted. During the interviews, we collected demographic data from participants through oral questioning.

Box. **The Interview Guide**

This study used semistructured questions during in-depth interviews. Following are examples of the open-ended questions posed to participants:

- Sample probes throughout: can you tell me a little more about your experience of _________; you mentioned ________, can you elaborate more on this?
- Please recall and describe an instance when you informed bereaved family members (particularly parents) of the death of a newborn.
- Before informing the family, how did you decide who should receive the news? What were your thoughts and feelings at the time?
- During that specific conversation, when you needed to convey the core message that “the child cannot survive” or “the child has died,” how did you deliver the news? What were your feelings at the time?
- In your experience communicating the news of a newborn’s death to family members, did cultural factors or social customs influence the communication process? Can you describe a specific moment when you felt this was the case? How did this influence your communication style at the time?
- Looking back on that specific communication experience, what aspects did you pay particular attention to during your conversation with the parents? What made you feel these aspects were important?
- When communicating information about the death of a child, have you ever noticed or confirmed whether the parents truly understood what you were saying (including medical terminology)? How did you perceive the parents’ level of understanding?
- When discussing the handling of the child’s remains, can you describe a specific conversation or interaction? How did you guide or participate in this process? How did you perceive the parents’ state of mind and needs during this process?
- In that specific communication experience, what kind of support do you feel you provided to the parents?
- Regarding health care providers’ experiences of communicating with parents in neonatal death events, are there any important aspects that I have not asked about that you feel are worth sharing?

### Data Analysis

Data were collected from September 2024 to May 2025 and analyzed from October 2024 to June 2025. Interview data were analyzed using the Colaizzi 7-step phenomenological approach.^29^ This analysis involved 4 researchers (H.Z., C.H., J.G., and P.Z.) repeatedly reading transcripts for familiarization, immersion, and understanding and identifying important statements related to NICU staff communication with families about neonatal end-of-life issues. Meaningful segments were extracted through team discussion, organized into formulated-meaning units, and clustered into subthemes and overarching themes. These themes were then integrated into a comprehensive description of the phenomenon, ensuring clear linkages to the core research focus. We purposefully selected 8 participants with diverse and representative work experience, and then we presented the derived thematic structure and descriptions to these participants for validation. These participants confirmed the accuracy and resonance of the findings. We analyzed the data in Chinese and translated the results into English.

The rigor of this study was assessed using the Lincoln and Guba criteria.^30^ We reread and deeply immersed ourselves in the participants’ narratives. We used verbatim quotations to illustrate the participants’ views. Two interviewers (H.Z. and J.G.) had no prior knowledge of or interaction with participants before data collection. When analyzing data, we adopted a stance of bracketing and temporarily set aside personal opinions and experiences, opening ourselves as much as possible to return to the participants’ descriptions of their experiences.^31,32^ To address discrepancies, our team held meetings in which members with differing views revisited the original interview transcripts, grounding their interpretations in thematic statements. Through discussion and reflection, consensus was ultimately achieved.

## Results

A total of 24 individuals (10 physicians and 14 nurses; mean [SD] age, 39.2 [7.5] years; 20 females [83%], 4 males [17%]) participated in the study, with 100% response rate (Table 1). Of the participants, 20 (83%) were married with at least 1 child, and the mean (SD) length of health care service was 16.3 (8.1) years. All participants held a bachelor’s degree, were of Han Chinese ethnicity, and reported no religious affiliation. The interview data were categorized into 3 main themes and 9 subthemes, which were supported by illustrative quotations (Table 2, Figure).

**Table 1.** Characteristics of Participants (N = 24)^a^

Sex	Age, y	Highest educational level	Length of health care service, y	Marital status	No. of offspring
Female	30-39	University degree	11-15	Single	0
Female	60-69	University degree	36-40	Married	1
Male	40-49	University degree	16-20	Married	1
Male	40-49	University degree	21-25	Married	1
Female	30-39	University degree	11-15	Married	1
Female	30-39	University degree	11-15	Married	1
Male	30-39	University degree	11-15	Married	1
Female	30-39	University degree	11-15	Married	1
Female	40-49	University degree	16-20	Married	1
Male	40-49	University degree	16-20	Married	2
Female	30-39	University degree	6-10	Single	0
Female	30-39	University degree	11-15	Married	1
Female	30-39	University degree	11-15	Married	1
Female	30-39	University degree	11-15	Married	1
Female	50-59	University degree	26-30	Married	1
Female	50-59	University degree	31-35	Married	2
Female	30-39	University degree	11-15	Married	2
Female	30-39	University degree	6-10	Single	0
Female	30-39	University degree	6-10	Married	1
Female	30-39	University degree	11-15	Married	1
Female	30-39	University degree	11-15	Married	1
Female	40-49	University degree	26-30	Married	1
Female	30-39	University degree	11-15	Married	1
Female	30-39	University degree	6-10	Single	0

^a^ Participant confidentiality was safeguarded by using data ranges when possible. All participants were of Han Chinese ethnicity and had no religious affiliation.

**Table 2.** Main Themes and Subthemes and Representative Quotes From the Interviews

Themes and subthemes	Quote
**Fathers were prioritized over mothers as to whom to make the death disclosure **	
Perceiving the father as the primary family decision-maker (Paternal relatives were prioritized as the primary notification recipients, with the father being the preferred choice for death disclosure.)	“You could tell the father was calling the shots just by listening to family conversations. When the child was first admitted, we’d discuss treatment plans with relatives. Most families would say, ‘Let the father decide.’”
	“Throughout hospitalization, I communicated exclusively with the father. Whether starting new medication or ordering tests, he signed all consents. That’s how I knew he was the decision-maker at home. In cases of poor prognosis with no treatment benefit, I’d approach the father first.”
	“When the child was hospitalized, all family members except the mother would come. After our discussions, it was clear the father was in charge. He’d ask all the questions while others just listened silently.”
Encountering barriers to direct death disclosure to the mother (Traditional childbirth beliefs, mandating that mothers observe a period of confinement [*zuoyuezi* in Mandarin], mostly prevented their participation in communicating medical details and/or decision-making. Concurrently, mothers were perceived as vulnerable and emotionally fragile, rendering them unsuitable recipients for death disclosure.)	“We rarely saw mothers. Most of them were confined at home doing ‘*zuoyuezi*’ [postpartum recovery]. They couldn’t come to the hospital.”
	“I didn’t have a chance to communicate with mom. A lot of children’s fathers would take into account that mom was more vulnerable at this time and he wouldn’t let her come to the hospital.”
	“Postnatal mother was fragile and emotionally unstable. I am concerned that informing the child’s mother of the child’s death may cause her harm. Thus, I avoided it.”
Being instructed to prioritize death disclosure to the father (Family members and the father himself explicitly instructed the health care practitioner to disclose the information to the father first, delegating the responsibility of subsequent communication to him.)	“Hospital protocol required two contact numbers. We explained that we primarily called the first contact. Overwhelmingly, families designated the father and explicitly told us: ‘Contact Dad for everything.’”
	“When registering for family information, many fathers would say that if my child’s condition changed, you must call me first, not mom.”
**Death disclosure was handled sensitively, with the explicit term *death* avoided**	
Incrementally disclosing bad news (Practitioners used a tiered disclosure approach with escalating terminology, moving from *critically ill* to *low probability of survival* to *not going to make it*. This phased information release mitigated emotional shock by allowing gradual comprehension. This approach aligns with cultural practices in regions in China such as Jiangsu and Zhejiang, where sequential notification [*critical condition* followed by *demise*] remains customary.)	“The death of a child was very painful for the family. We had to tell the family step by step about the death of a child.”
	“We told…the relatives at every stage of the disease progression. We’d start by reviewing the child’s entire condition. Then, we tell the dad that the child was critically ill and the chances of survival were slim. Finally, we told the relatives that the child’s final outcome might be death.”
Avoiding directly using death-related vocabulary (Within Chinese cultural contexts, clinicians actively circumvented explicit terms such as *death*. Instead, they substituted culturally sanctioned euphemisms such as *not going to make it* to convey the finality of death indirectly.)	“We said ‘the child was not going to make it’ or ‘had found relief’ instead of ‘died.’”
	“For younger parents, colloquial terms like ‘gua le’ [death in local language] were used. That’s a more witty and youthful way of expression.”
	“I never said things like the child was dead. I just said I did the best I could and there was nothing more I could do for this kid.”
	“I wouldn’t use the word ‘death.’ That’s too cold-blooded an action for me to take.”
Using metaphors to explain medical terminology (Complex medical realities were translated through deliberate metaphorical framing. The battery-machine analogy reduced life to a functioning device, conceptualizing death simplistically as a component failure or mechanical cessation.)	“Previously I met a child who was brain dead and there was no point in treating him anymore. When talking to the family about the cause of the disease, I used the example of a machine running. The child was the machine and the brain was the battery. When the battery [had] gone irreversibly bad, the machine didn’t work. Likewise, the child passed away.”
	“This child had ARDS and was on a ventilator. I compared the child’s lungs to a balloon. A normal balloon could be filled up with a little effort. But this child’s lungs were like a torn balloon, they can’t be blown up at all. The child couldn’t breathe on his own. If we removed the ventilator, he would die.”
**Postdisclosure procedures were handled exclusively in accordance with cultural norms**	
Suppressing personal expressions of grief (The clinician’s emphasis on greater rationality embodies the Confucian principle of the golden mean. Excessive manifestations of grief may be perceived as disrupting social harmony, especially in the case of a perceived unnatural death. In this context, mean equates to maintaining inner equilibrium before emotions arise and observing ritual propriety in their expression, thereby avoiding extremes.)	“When I first encountered [a] little baby dying, I was really upset. There were other doctors and nurses comforting the family. I just went ahead and hid in the on-call room and cried for a while. I didn’t want the family to see me crying. It wasn’t appropriate.”
	“I was afraid to look [at] the families. I was afraid that if I watched them crying in pain… I would cry too. Sometimes I turned my back and wiped my tears.”
	“I was sad, but I didn’t act sad in front of the families. I felt that I communicated this matter with the family in a calm tone, and it caused the least harm to the family. Displaying personal sadness might pressure families to grieve.”
Being delegated full authority for handling the child’s remains (Driven by traditional customs deterring home-based rituals, families frequently relinquished custody of the child’s remains. Health care staff were entrusted with complete responsibility for disposition of the remains.)	“The tradition on our side is that we can’t take children’s remains home. We respect the tradition, but we can’t force the family to ask for the child’s remains to be taken away. But in my entire career, no family asked to take a child’s remains home. The family left after signing a consent form for the disposal of the remains. It’s up to us to do the rest.”
	“The death of a small infant is a bad luck event, and bringing home the remains of a child for a ceremony to mourn is also a bad thing. Therefore, most families would not take the body of a child home for burial. Instead, they signed a consent form and entrust us with the cremation of their child’s body.”
	“We would pack up the [personal artifacts] from the child’s previous use in the hospital and gave them to the family. But the families wouldn’t take it. They told us to do with it as we please and not to give it to them.”
Adjusting disposition arrangements to safeguard familial reputation (Neonatal death is traditionally categorized as an inauspicious event, potentially inviting social scrutiny and damaging family “face.” Consequently, disposition arrangements were often modified by the health care team to mitigate reputational harm for the parents.)	“Some families asked us to take their child’s body to the morgue at night. The family felt that the hospital is less crowded at night so that not too many people could see them delivering the body and [they] couldn’t know that their child has died.”
	“Some families asked that the child’s remains be sent to the morgue in a box. They said they didn’t want anyone to see that it was a little baby being sent to the morgue. We usually put the body in a red bag or a cardboard box and sent it to the morgue.”
	“There was always a gathering of people at the entrance to our section. The families of the dead child didn’t want us to be seen delivering the remains. They asked us not to use the main entrance of the department. Then, we took the staff corridor to deliver the body.”

Abbreviation: ARDS, acute respiratory distress syndrome.

**Figure.**
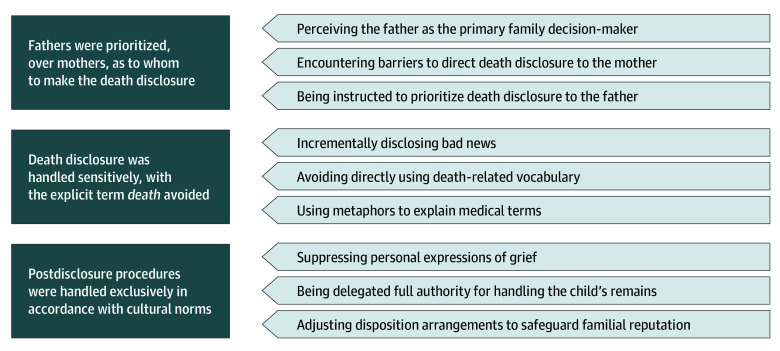
Schematic of Health Care Workers' Experiences of Communicating With Family Members Regarding the Death of a Neonate

### Theme 1: Fathers Prioritized Over Mothers As to Whom to Make the Death Disclosure 

The interviews revealed that NICU staff perceived the father as the primary family decision-maker. One participant stated, “You could tell the father was calling the shots just by listening to family conversations.” Another participant noted, “Throughout hospitalization, I communicated exclusively with the father.” This observation highlights the father’s dominant decision-making role within the family. Another participant admitted, “After our discussions, it was clear the father was in charge.”

Medical staff encountered barriers to direct death disclosure to the mother. In China, women are expected to observe *zuoyuezi* following childbirth. Two participants said, “We rarely saw mothers. Most of them were confined at home doing ‘*zuoyuezi*.’” Another participant stated, “I didn’t have a chance to communicate with mom...wouldn’t let her come to the hospital.” Furthermore, the staff took into account that the mother was physically weak. They stated, “I am concerned that informing the child’s mother of the child’s death may cause her harm. Thus, I avoided it.”

Medical staff were instructed by family members to prioritize death disclosure to the father. We found men and families imposed paternalistic decision-making on women, limiting women’s access to medical care and associated decision-making. One participant said, “Overwhelmingly, families designated the father and explicitly told us [to] contact dad for everything.” Another clinician stated, “Many fathers would say that...you must call me first, not mom.”

### Theme 2: Death Disclosure Handled Sensitively, With Term *Death* Avoided

Physicians incrementally disclosed bad news. One participant admitted, “We’d start by reviewing the child’s entire condition. Then, we told the dad that the child was critically ill and the chances of survival were slim. Finally, we told the relatives that the child’s final outcome might be death.” Similarly, another participant said, “The death of a child was very painful for the family. We had to tell the family step by step about the death of a child.”

All participants avoided directly using death-related vocabulary in their disclosures. As one participant noted, “We said ‘the child was not going to make it’ or ‘had found relief’ instead of ‘died.’” Another clinician said, “I wouldn’t use the word ‘death.’ That’s too cold-blooded an action for me to take.”

Physicians also used metaphors to explain medical terminology. One participant made this analogy, “The child was the machine and the brain was the battery. When the battery [had] gone irreversibly bad, the machine didn’t work. Likewise, the child passed away.” Another participant stated, “But this child’s lungs were like a torn balloon, they can’t be blown up at all. The child couldn’t breathe on his own. If we removed the ventilator, he would die.”

### Theme 3: Postdisclosure Procedures Handled Exclusively in Accordance With Cultural Norms

Medical staff mostly suppressed personal expressions of grief. One participant mentioned, “I was afraid to look at the families. I was afraid that if I watched them crying in pain…I would cry too.” Another participant stated, “I was sad, but I didn’t act sad in front of the families.... Displaying personal sadness might pressure families to grieve.”

A particularly noteworthy finding was that medical staff were often delegated full authority for handling the newborn’s remains. As one participant described, “The family left after signing a consent form for the disposal of the remains [by the hospital]. It’s up to us to do the rest.” Another participant also stated, “We would pack up the [personal artifacts] from the child’s previous use in the hospital and gave them to the family.... They told us to do with it as we please and not to give it to them.”

Additionally, medical staff would adjust disposition arrangements to safeguard familial reputation. Because neonatal death is often regarded as inauspicious in China, families may face gossip or societal judgment. Most families preferred private, discreet handling of the newborn’s body and related arrangements. One participant noted, “Some families asked us to take their child’s body to the morgue at night...hospital is less crowded at night...not too many people...[they] couldn’t know that their child has died.” Another participant stated, “[Family members] said they didn’t want anyone to see that it was a little baby being sent to the morgue. We usually put the body in a red bag or a cardboard box and sent it to the morgue.” Another clinician added, “[Family members] asked us not to use the main entrance of the department. Then, we took the staff corridor to deliver the body.”

## Discussion

Our findings indicated that fathers were prioritized over mothers to be the recipient of the death disclosure. This theme reflects the profound influence of patriarchal society within Confucian culture.^17,33,34^ In China, traditional family systems are patriarchal, characterized by male-dominated decision-making and subordinate female status.^17,18^ Although the Cultural Revolution of the 20th century challenged these values, male dominance still exists.^35,36^ Therefore, we found that health care practitioners perceived fathers as the primary family decision-maker. Similarly, collectivism maintains that family as a whole, rather than any individual member, has ontological priority and that collective decision-making is required on vital matters.^14^ Therefore, disclosing medical information is perceived as a family-centered process.^14,15,16^ These cultures empower senior family members to make decisions on behalf of younger ones.^14,37^ In our study, medical personnel were instructed to prioritize death disclosure to the father.

Participants also reported encountering barriers to direct death disclosure to the mother. Specifically, they rarely encountered mothers in the hospital and had limited opportunity to communicate with them. In China, traditional childbirth beliefs mandate mothers to stay at home for 30 to 40 days to recuperate (*zuoyuezi*).^7^ Consequently, most mothers are not present in hospitals. Furthermore, traditional culture dictates that mothers remain emotionally stable and not cry.^38,39^ However, a child’s death inflicts great trauma to the mother. Therefore, in our study, most mothers were not directly informed by physicians of their newborn’s death. This news was instead conveyed by the family.

A key finding was that death disclosure was handled sensitively, with the medical staff avoiding using the explicit term *death*. This disclosure strategy involved incrementally communicating bad news, avoiding direct death-related vocabulary, and using metaphors to explain medical terminology. This pattern reflects both legal and cultural components: although laws and regulations mandate telling patients and families the truth, they also oblige health care professionals to prevent the potential adverse consequences of that truth.^12,40,41^ Meanwhile, death and public dialogue about death are taboo in traditional Chinese culture.^13,42^ The vast majority of Chinese people believe that openly discussing (even merely thinking about) death or dying brings bad luck.^43,44^ Another study showed that mentioning death in traditional Chinese culture is considered disrespectful and even blasphemous.^45^ Thus, in the present study, while adhering to legal and cultural norms, the participants exercised caution in disclosing death and avoided direct mention of the word *death*. Euphemisms that function metaphorically or metaphors are widely used in the field of clinical communication.^46,47^ By visualizing abstract concepts such as death through a system of metaphors, health care professionals are balancing risks, benefits, and individual needs as a means to reducing the perceived impact of communicating details at the end of life. In this study, the participants used metaphors to interpret medical terminology, thereby concretizing the concept of neonatal death.

The Analects and The Doctrine of the Mean are foundational Confucian texts. The Analects advise, “Grieve, but not to the point of self-injury; rejoice, but not to the point of excess.”^48^ This text advocates maintaining inner equilibrium before emotions arise and observing ritual propriety in expression, thereby avoiding extremes.^49^ A similar idea is articulated in The Doctrine of the Mean.^50^ Following Confucianism, people should not be seen expressing their emotions, such as sadness or grief, in public.^51^ In this study, health care practitioners’ suppression of their emotions was a product of Confucian emotional culture norms.

Participants were granted full authority to handle the remains of newborns. In Western countries, through the memorial service, parents can acknowledge the child’s existence and express their grief.^52^ In contrast, Chinese cultural practices focus on facilitating the mother’s separation from the deceased child and the associated events.^53^ This phenomenon is similarly found in some Southeast Asian countries.^54^ Maintaining a connection to a stillborn child is believed to not only potentially hinder the family’s ability to move forward but also obstruct the deceased newborn’s journey toward reincarnation.^7^ Our participants reported that, after signing the commitment or consent statement, family members discontinued any further involvement. Conversely, the absence of death rituals limits parents’ participation in culturally meaningful practices crucial for processing grief.^55,56,57^ Further research is needed to determine whether Chinese families experiencing neonatal death wish to alter this tradition. Additionally, the medical personnel in our study adjusted disposition arrangements to safeguard familial reputation. In China, neonatal death is viewed as a contagious misfortune and a moral failing of the family, leaving families potentially facing social isolation.^58^ Simultaneously, these families may endure societal judgment.^57,58,59^ Confucian values emphasize the importance of family continuity and good death.^60^ As a result, the neonatal death becomes reframed as a form of punishment directed at the mother, who then bears additional shame due to others’ suspicions.^59^ Given these cultural beliefs, health care professionals carefully considered the potential stigma and arranged for the discreet management of neonates’ remains to protect the family from public scrutiny.

### Limitations

This study has some limitations. First, Chinese culture varies across regions. The sample in this study was from Jiangsu Province only, which limited the generalizability of the results. Second, we used a purposive sampling strategy to maximize participant diversity. However, participation in qualitative interviews was voluntary, thus creating a possible selection bias. Third, this study focused on the experiences of medical staff, and the experiences of family members were not analyzed. This focus might limit a deeper understanding of clinician-patient communication.

## Conclusions

In this qualitative study of death disclosure, participating NICU physicians and nurses disclosed death in accordance with cultural norms and with sensitivity. Under practical constraints, participants prioritized informing the father of the newborn’s death. During disclosure, they used metaphors and avoided using death-related vocabulary to gradually inform the parents. Additionally, participants restrained their emotions and handled the disposition arrangements to protect the family’s reputation. These findings highlight key areas for improvement such as reducing stigma around neonatal death through greater public awareness; developing culturally sensitive hospital bereavement services; and exploring parents’ experiences with communication about neonatal deterioration and death, preferences for disposition of remains, and interest in hospital-based mourning or remembrance ceremonies.

## Notes

Supplement.
Data Sharing Statement
