# Supplementary material for: Culturally Informed Communication of Neonatal Death in Chinese Neonatal Intensive Care Units
Source: JAMA Netw Open. 2026 Apr 9;9(4):e265919. doi: 10.1001/jamanetworkopen.2026.5919 (PMC13067010; doi:10.1001/jamanetworkopen.2026.5919)
Supplement: Supplement. — Data Sharing Statement [file jamanetwopen-e265919-s001.pdf]

Data Sharing Statement

Zhao. Culturally Informed Communication of Neonatal Death in Chinese Neonatal Intensive Care Units. *JAMA Netw Open*. Published April 09, 2026.
doi:10.1001/jamanetworkopen.2026.5919

Data
Data available: Yes
Data types: Deidentified participant data
How to access data: How to access data: Please reach out to the corresponding author of this study Dr. Pingting Zhu to request data sharing: ptzhu@yzu.edu.cn
When available: With publication

Supporting Documents
Document types: None

Additional Information
Who can access the data: researchers whose proposed use of the data has been approved
Types of analyses: for a specified purpose
Mechanisms of data availability: with a signed data access agreement
